# Functional and Quality Characteristics of Ginger, Pineapple, and Turmeric Juice Mix as Influenced by Blend Variations

**DOI:** 10.3390/foods10030525

**Published:** 2021-03-03

**Authors:** Akama Friday Ogori, Julius Amove, Precious Aduloju, Giacomo Sardo, Charles Odilichukwu R. Okpala, Gioacchino Bono, Małgorzata Korzeniowska

**Affiliations:** 1 Department of Home Sciences, Faculty of Agriculture, Federal University Gashua, Gashua P.M.B.1005, Nigeria; ogorifaraday@gmail.com; 2 Department of Food Science and Technology, Federal University of Agriculture Makurdi, Makurdi 970211, Nigeria; amovejulius@yahoo.com (J.A.); preshus06@gmail.com (P.A.); 3 Institute for Biological Resources and Marine Biotechnologies (IRBIM), National Research Council of Italy (CNR), 91026 Mazara del Vallo, Italy; giacomosardo88@gmail.com (G.S.); gioacchino.bono@cnr.it (G.B.); 4 Faculty of Biotechnology and Food Science, Wrocław University of Environmental and Life Sciences, 51-630 Wrocław, Poland; malgorzata.korzeniowska@upwr.edu.pl

**Keywords:** ginger, pineapple, turmeric, juice mix, physicochemical properties, microbiological quality, sensory attributes

## Abstract

In this current work, the functional and quality characteristics of ginger, pineapple, and turmeric juice mix as influenced by blend variations were investigated. Specifically, the blends had constant ginger amounts, decreased pineapple, and increased turmeric proportionally. Additionally, the functional properties involved physicochemical (pH, soluble solids (SS), total titratable acidity (TA) and viscosity), proximate (moisture, protein, fat and ash), minerals (Ca, and Mg) and vitamin C and β-carotene analyses, whereas quality properties involved microbiological and sensory analyses. The results showed that as quantities of pineapple and turmeric respectively decreased and increased, there was significant increases in Ca, Mg, vitamin C, and β-carotene contents (*p* < 0.05). Across the blends, the degree of significant differences (*p* < 0.05) in the protein, fat, and ash seemed more compared to those of moisture contents. Despite the increases in pH and viscosity, and decreases in SS and TA, the increases in turmeric potentially reinforced by ginger most likely decreased the bacterial/fungi counts, as well as inhibition zones. Increasing and decreasing the respective amounts of turmeric and pineapple might not necessarily make the blends more acceptable, given the decreases in appearance, taste, aroma, and mouthfeel scores.

## 1. Introduction

Broadly, fruits can be grouped into two categories, namely: dry and fleshy/succulent fruits, and this is largely based on the physical ripe condition [1]. When properly harvested, fruits like orange, pineapple, and watermelon are edible, fleshy, and sweet [2]. Processing of fruit involve enzymes, extraction, and evaporation activities. Additionally, the suitability of a fruit juice and its concentrate/extract for an intended application remains dependent on its quality [1]. Fruits endocarps and mesocarps contain various phytochemical compounds resembling vegetables, with higher amounts of free waters, but lower amounts of carbohydrate, fat, and protein [3]. When the natural liquid of freshly harvested fruit like orange is squeezed, a juice drink is produced and is available for immediate consumption [1]. The regular consumption of fruits and its juices, most importantly, helps to make up for diet nutritional losses as well as maintain health and wellbeing [4]. Anticipating how the freshness of fruit (as well as vegetable) quality in the form of juice drink would continually keep remains challenging [1]. 

The relatively high metabolic activity in fruits like apple, banana, and pineapple, for instance, continues even after harvesting, which makes them highly perishable [5]. Among the above-mentioned fruits, pineapple (*Ananas comosus*) stands unique because it is among the few bromeliads that produce edible fruits, with morphologically fused berries around a central core [6]. Pineapples comprise antioxidants/polyphenolic compounds, natural enzymes, and pro-vitamins [4,7]. Specifically, a ripe and ready-to-harvest pineapple would have (above-mentioned) berries comprise bioactive and phenolics contents/non-toxic compounds, which presents promising therapeutic potentials that help to enhance immune response [6]. Essentially, the fleshy and juicy pulp makes pineapple an excellent blend to obtain new flavours in beverages and juice mixes. Moreover, mixed juice blends produced from various fruits can help combine basic nutrients and provide improved nutritional value [4].

Ginger (*Zingiber officinale* Rosc) is an underground rhizome or stem of herbaceous perennial species of family Zingiberaceae, also considered typically indigenous to many tropical/subtropical countries [8,9,10]. As a widely established monocotyledon herb, the main products of ginger include dry or fresh rhizome, as well as ground ginger (powder) [9,10,11]. It can also be used as a whole juice extract and in drink/tea after blending process [12]. The rhizome/stem of ginger, in addition to comprising such proximate components like ash, carbohydrate, fiber, moisture, and protein, has volatile oil of stem that contributes to provide its pleasant aroma [10,13,14,15]. Additionally, ginger contains ascorbic acid, β-carotene, curcumin, gingerol, linalool, paradol, γ-terpinene, as well as terpinen-4-ol [10,16,17,18,19]. The swollen rhizome/stem of ginger has been associated with antimicrobial, anti-inflammatory, and anti-carcinogenic properties [12]. 

Turmeric (*Curcuma longa* Linn.), equally an underground rhizome like ginger, and within the family of Zingiberaceae, is largely available either in dry or fresh forms [10,20]. Turmeric, largely cultivated across warm climatic regions of the globe, serves as a common food additive mostly in powdered form. Turmeric (powdered), positioned as a colorant, can serve as a flavouring agent in food formulations [21,22]. Commonly grown in many parts of Nigeria, the production of turmeric has made its sales provide economic and regional benefits [23]. To convert turmeric into a stable commodity, there is need for a number of processing operations, which includes boiling, cleaning, slicing, curing, drying, grading, milling, and packaging [24]. For emphasis, turmeric not only fortifies the drinks that it is added to, it is also able to improve the nutritional quality [23]. Besides its role as spice, food preservative, and coloring material, turmeric occupies a space in traditional medicine given the many scientific studies that revealed its many bioactivities like anti-inflammatory, anti-bacterial, anti-carcinogenic, anti-diabetes, and antioxidant capacities [25]. Largely, turmeric comprises 60% turmerone, 25% zingiberene, and 1.5–5% volatile oil. In particular, turmeric comprises three curcuminoids, namely: bisdemethoxycurcumin (0.30–9.10%), curcumin (diferuloylmethane) (71.50–94%), and demethoxy-curcumin (6–19.4%), which cumulates to the curcuminoids (2.5–8%) that bring about the yellow coloration [10,26,27]. 

Blending spices with fruits to form a juice mix is becoming increasingly popular in Nigeria, with high promise of spreading to the West Africa sub-region. Additionally, there is increasing notion among many that ginger, pineapple, and turmeric juice mix is affordable, nutritionally enriching, as well as filling, and this is yet to be scientifically verified. To our best knowledge, the blend variations of ginger, pineapple, and turmeric juice mix has not been studied. It is anticipated that a juice mix of this type could result in a nourishing composite with promising functional and sensory qualities. To supplement existing information, the aim of this current study was to determine the functional and quality characteristics of ginger, pineapple, and turmeric juice mix as influenced by blend variations. Specifically, the functional properties involved minerals and vitamins, physicochemical, and proximate components, whereas quality properties involved microbiological and sensory components.

## 2. Materials and Methods

### 2.1. Overview of Experimental Program

**Figure 1:**
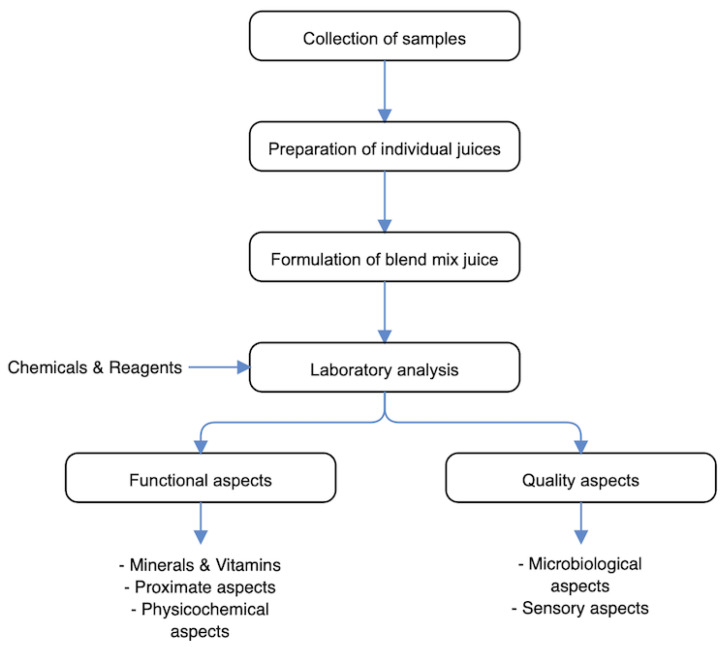
The schematic overview of the experimental program, depicting the essential stages from the collection of ginger, pineapple and turmeric, and preparation of individual juices, to the formulation to make the mix juice, and then, the functional and quality aspects of the laboratory analyses.

The schematic overview of the experimental program, depicting the essential stages from the collection of ginger, pineapple and turmeric, and preparation of individual juices, to the formulation to make the mix juice, and then, the functional and quality analyses, is given in Figure 1. For emphasis, this current study specifically targeted determining the functional and quality characteristics of ginger, pineapple, and turmeric juice mix as influenced by blend variations. Specifically, the functional properties involved minerals and vitamins and physicochemical and proximate components, whereas quality properties involved microbiological and sensory components. The end goal is to achieve a juice mix that could bring about a nourishing composite with promising functional and sensorial attributes.

### 2.2. Collection of Samples

The ripe pineapple, matured turmeric, and ginger were purchased from the Railway (7.72732° N, 8.53193° E) and Wadata (7.74527° N, 8.51339° E) markets situated in Makurdi, Benue State, Nigeria. All samples were taken to the laboratory for sample preparation and analysis.

### 2.3. Chemicals and Reagents

All the chemicals and reagents utilized in this current study were reagent grade standard.

### 2.4. Preparation of Pineapple Fruit Juice

**Figure 2:**
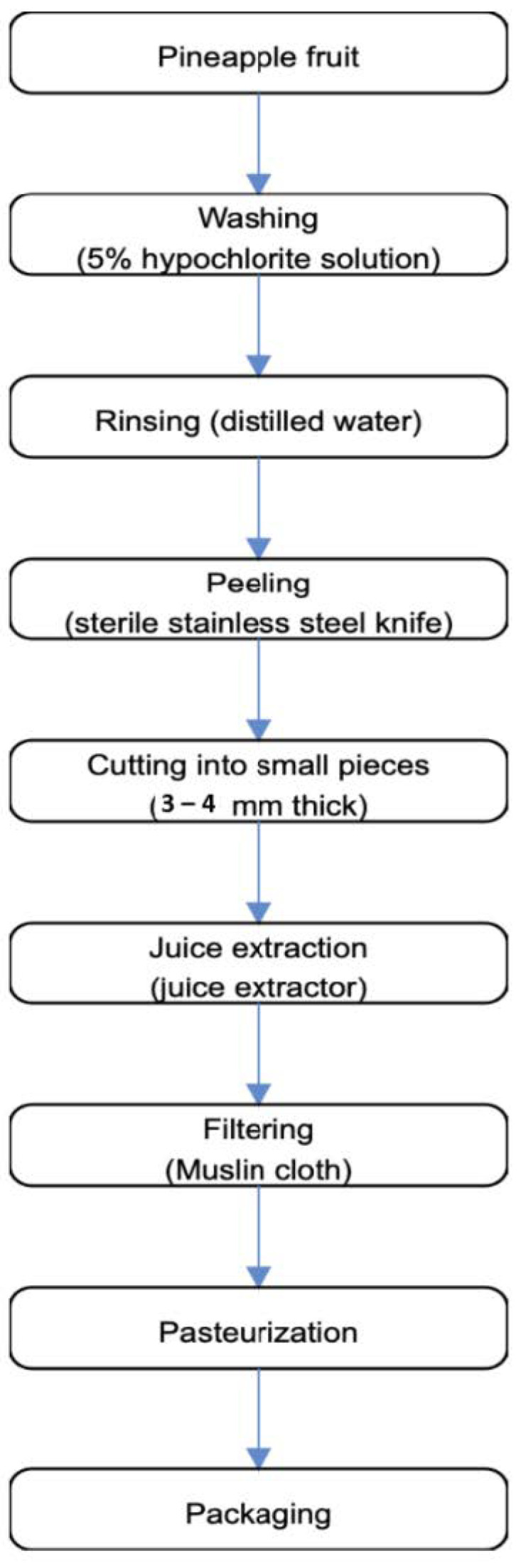
The preparation of pineapple fruit juice (Okwori et al., [28]).

The preparation of pineapple fruit juice followed the method of Okwori et al. [28] with slight modifications, depicted in Figure 2. Pineapple fruits were selected and washed with 5% HOCl solution and thoroughly rinsed with distilled water before peeling with a sterilized knife. The fruits are cut into sizes of about 3–4 mm thick and juice extraction using a juice extractor. The pineapple juice was filtered using sterile muslin cloth, which was folded into two layers and filtered into a clean transparent bowl. The juice was filled into an air-tight screwed cap, pasteurized, and refrigerated at ~4 °C prior to analysis.

### 2.5. Preparation of Turmeric and Ginger Juice

**Figure 3:**
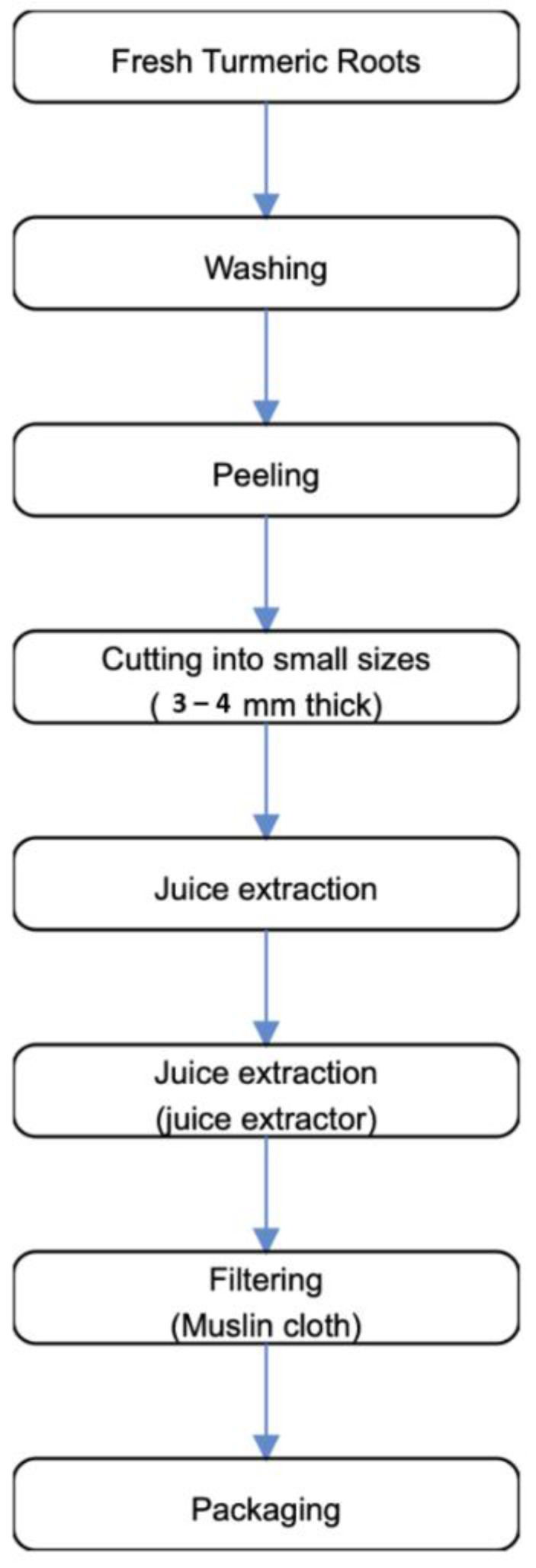
The preparation of turmeric juice (Source: Top 10 Home Remedies Team [29]).

Following the method prescribed by the Top 10 Home Remedies Team [29], herein depicted in Figure 3, five fresh turmeric rhizomes were rinsed under clean running tap water to remove the dirts. The turmeric rhizomes were peeled and then cut into pieces and put into the blender, and at the same time, supplemented little equivalents of clean/filtered water were added to ease friction during blending. The juice pulp was then filtered using a sterile muslin cloth to get the juice, which was subsequently refrigerated at ~4 °C, until required.

The preparation of ginger juice is similar to that of turmeric juice. Fresh ginger roots were washed under clean running tap water, peeled, and then cut into smaller pieces, thereafter, they were subjected to blending, and at the same time, supplemented with little amounts of clean/filtered water to ease friction during blending. The juice pulp was then filtered using a sterile muslin cloth to get the juice, which was then refrigerated at ~4 °C, until required.

### 2.6. Formulation of Pineapple, Turmeric and Ginger Blend Juice Mix

The formulation of pineapple, turmeric, and ginger blend juice mix is given in Table 1. The juice from pineapple, turmeric, and ginger juices were blended at varied proportions. This followed the method demonstrated by local artisans, but with slight modifications to enable reproducibility. Specifically, the control sample was pineapple only, that is, PJ:TJ:GJ = 100:0:0. The blends kept the ginger amounts constant, decreased the pineapple and increased the turmeric amounts by proportion. Next, the juice blends were mixed by stirring, bottled with screw caps before the pasteurization at 65 °C for 5 min, in a thermostatically controlled water bath, and thereafter, cooled at ambient temperature of about 27 °C. At the end, the blend juice mix samples were refrigerated at ~4 °C until required for analysis.

### 2.7. Functional Analysis.

#### 2.7.1. Minerals and Vitamins Measurements

##### Determination of β-Carotene 

The β-carotene of samples was determined using the AOAC method [30]. About 5 g of the sample was transferred into a separating funnel and a solution containing 60 mL of hexane; 40 mL of ethanol were swirled vigorously after adding 2 mL of 2% NaCl. This was then allowed to stand for 30 min after which the lower layer was discarded. The absorbance of the top layer was determined at a wavelength of 460 mm using a spectrophotometer, using the equation below:

$$TC=\frac{absorbance}{100\;specific\;extinction\;\times \;pathlength\;of\;the\;cell}$$

where,

TC: Total carotenoids (mg)

Molar extinction coefficient (∑) = 15 × 10^−4^

Specific extinction coefficient (∑) = (∑ × molar mass of β-carotene)

Molar mass of β-carotene = 536.88 g/mol

Path length of cell = 1 cm

##### Determination of Calcium and Magnesium

The mineral composition (specific to Ca and Mg) of samples were determined by AOAC acid digestion method [31]. Ash obtained after incineration at 600 °C was dissolved in 5 mL HCl solution and transferred into a 50 mL volumetric flask. The resulting solution was made to mark with distilled water. The mineral contents were then measured using atomic absorption spectrophotometer (AAS), and mineral composition results were recorded.

##### Determination of Vitamin C

The vitamin C of samples was determined using the method described by Ikewuchi and Ikewuchi [32]. The quantities of vitamin C present are measured by the tiny additions of acidified starch (termed “reaction mix”), followed by droplets of iodine until purple color. Any vitamin C will “neutralize” the iodine, to prevent the purple color formation. In line with this, iodine solution (0.1 M) was prepared using 10 g of KI, and starch solution, using 0.25 g of starch powder. In order to actualize the vitamin C, a blank solution (25 mL) was made from the sample, and 10 drops of starch solution were added. The mixture was titrated with iodine solution until the first black blue color, which persisted for ~20 s. Blended juice samples (25 mL) were titrated exactly the same way as the standard solution. The initial and final volume of iodine solution required to produce the color change at the end points were recorded. Subsequently, the vitamin C concentration was determined as follows:

vitamin C concentration in the juices (g/100 mg) = y/b

where 

b = titre (mL) from the titration of the standard vitamin C solution

y = titre (mL) from the titration of the sample solution.

#### 2.7.2. Proximate Measurements 

##### Determination of Moisture 

The moisture of samples is determined by the AOAC method [31]. Cleaned crucible is dried in the oven at 100 °C for 1 h to constant weight and then cooled in the desiccator. Approximately 2 g of the samples were weighed into the crucible and dried at 100 °C to a constant weight, and calculated as below:

$$\%Moisture=\frac{Weightloss\;\times \;100\%}{Weight\;of\;samples}$$

##### Determination of Crude Protein 

The crude protein of samples was determined using the AOAC method [31] with slight modifications. Approximately 1 g of the sample was placed with a selenium catalyst in the micro Kjeldahl digestion flask. The mixture was digested to clean clear solution. The flask was cooled and then diluted with distilled water to the 50 mL mark of a conical flask, 5 mL of the mixture was transferred into distillation apparatus, and 5 mL of 2% boric acid added unto 100 mL conical flask (the receiver flask) with four drops of methyl red indicator. Then, 50% of NaOH was constantly added to the digested sample until the solution turned cloudy, indicating the solution had achieved alkalinity. Distillation was carried out in the boric acid solution at the receiver flask. During the distillation process, the pink color of the solution in the receiver flask turned blue, indicating the presence of ammonia. The resulting solution in the conical flask was then titrated with 0.1 M HCl and the protein content calculated as below:

%Nitrogen×6.25 (1 mL of 0.1 NHCL=0.0014 gN)

$$Nitrogen=\frac{Titrevalue\;–\;blank\times 0.0014\;N\times 100\%\times 25}{Weight\;of\;sample\times 5\;mlaliquot}$$

##### Determination of Crude Fat

The crude fat of samples was determined using the AOAC method [31] with slight modifications. The 100 mL beaker used was washed and dried in an oven for 1 h at 105 °C, and thereafter cooled in a desiccator and weighed. Approximately 10 mL of the samples was mixed with hexane in a separating funnel, and the organic layer was transferred into the pre-weighed beaker, subject to water bath, and thereafter weighed. The crude fat was determined using the equation below:

$$\%crudelipid=\frac{Weight\;of\;the\;fat\;\times \;100\%}{Weight\;of\;the\;sample}$$

##### Determination of Ash

The ash of samples was determined from the loss in weight during incineration following the AOAC method [30] with slight modifications. This method allows the entire organic matter to be burnt off, without the appreciable decomposition of the ash constituent. Approximately 5 g of the samples were placed in the incinerator. The ashing was done at a furnace of 600 °C for 6 h and calculated as below:

$$Ash\;Content=\frac{Weight\;of\;ash\times 100\%}{Weight\;of\;the\;sample}$$

#### 2.7.3. Physicochemical Measurements

##### Determination of pH

The pH of samples was determined using a pH meter, calibrated with buffers standard. The electrode was rinsed with distilled water, the electrode was then dipped into 5 g of the sample, which had been dissolved in 50 mL of water.

##### Determination of Soluble Solids

The soluble solids of samples were determined using the AOAC method [30]. The prism of the refractometer was cleaned and a drop of the blended juice was placed on the prism and closed. The °Brix was read using the scale of the refractometer when held close to the eyes.

##### Determination of Titratable Acid (TA)

The titratable acid of samples was determined using the AOAC method [30] with slight modifications. Approximately 10 mL of the juice was pipetted into a conical flask and 25 mL of distilled water added to make a solution. Approximately 200 mL of 0.1 M of NaOH was titrated against the sample using phenolphthalein as an indicator, to achieve color pink as an end point. The corresponding burette reading was taken using the following formula:

$$TA=\frac{Titre\times blank\times Normality\;of\;base\times mlequivalent\;of\;citricacid}{Weight\;of\;Sample}$$

where, *TA* = titrable acidity (%)

##### Determination of Viscosity

The juice samples viscosity was determined using a Brookfield viscometer (model Lv-3, Middleboro, MA 02346, USA) with the spindle set at 60 rpm, after which the readings were recorded in millipascal-second (mPa.s).

### 2.8. Quality Analysis

#### 2.8.1. Microbiological Evaluation

Microbiological analysis of the juice mix was carried out following the method described by Adegoke [33], with slight modifications, following the pour-plate method. This enabled the determinations of total bacteria and fungi counts. Homogenized (~60 s) quantities of blend (~2 g) with 15 mL of diluents was prepared. Serial ten-fold dilution of homogenate involved 0.1 mL of aliquots aseptically introduced into sterile Petri dishes, after which molten agar (~45 °C) was poured unto them, mixed and then allowed to set. The different agar plates were incubated for ~24 h. Nutrient Agar (NA) was used for the enumeration of total bacteria count and was then incubated at 37 °C for 24–48 h. Sabourd Dextrose Agar (SDA) was used for the enumeration of total fungi count then incubated at room temperature (28 ± 2 °C) for 3–5 days. The microbiological analysis were reported in terms of logarithm of colony forming units (log cfu/mL) of the blend sample.

Antibacterial activity of the juice extracts was determined by molten Agar well diffusion technique following the method of Abubakar et al. [34] with slight modifications. The test organism (*Salmonella typhii*) was diluted with Muller Hinton broth to 0.5% McFarland equivalent standard. Approximately 25 mL of Mueller Hinton Agar (HiMedia) plates were checked for sterility and streaked with an overnight broth cultured of bacterial isolate, using sterile cotton buds. A standard sterile cock borer of 6 mm diameter was used to make uniform wells on the surface of the streaked agar media. With the aid of a micropipette, the wells were filled up with 200 µL each of the undiluted blended juice extract (sample A–E). The plates were then allowed to stand for ~1 h in the refrigerator to allow proper diffusion of the extract. Amoxycillin (~25 mg/mL) solution was prepared and served as the control [31]. Following the method of Rahman et al. [35], all the plates were incubated at 37 °C for ~24 h, after which the antibacterial activity was evaluated based on the diameters of zones of inhibition and recorded in millimeter (mm).

#### 2.8.2. Sensory Evaluation

The ginger, pineapple, and turmeric juice mix blends were subjected to sensorial evaluation. This was done with the help of 10 (*N* = 10) panelists, comprising of students and staff of the Food Science and Technology Department, Federal University of Agriculture Makurdi. Specifically, these panelists underwent sensorial training prior to their participation at this study. Importantly, the panelists’ participation was voluntary. Additionally and prior to their participation, the verbal consent was taken from all the panelists. To ensure privacy, gender was not indicated. The selection criteria was based on complete participation of sensory training for this study. The samples were presented in a white plastic cup to each panelist. Each sample presented was coded. Essentially, each panelist was provided with adequate space to ensure there was no co-operation during the sampling of juice mix blends. The sensory attributes comprised appearance, taste, aroma, mouthfeel, and general acceptability. Consistent with the method described by Iwe [36], the sensory attributes were individually considered based on a 9-point Hedonic scale, which had the least value (numeric value = 1) designated as ‘disliked extremely’, and the highest value (numeric value = 9) designated as ‘liked extremely’.

### 2.9. Statistical Analysis

One-way analysis of variance (ANOVA) was used to analyse the emergent data. The results were presented in terms of mean values ± standard deviation (SD) from duplicate measurements. The mean values were resolved with the help of Fisher’s Least Significant Difference (LSD). The probability level of statistical significance was set at *p* ˂ 0.05 (95% confidence interval). IBM SPSS software (version 22.0) was used to do the data analysis.

## 3. Results and Discussion

### 3.1. Functional Aspects Minerals and Vitamins Variations

The minerals and vitamins variations of ginger, pineapple, and turmeric juice mix as influenced by blends can be seen in Table 2. Clearly, significant differences (*p* < 0.05) in Ca, Mg, vitamin C, and β-carotene contents were found across samples. Specifically, the Ca, Mg, vitamin C, and β-carotene contents increased significantly (*p* < 0.05) as quantities of pineapple and turmeric were respectively decreased and increased. The control PJ:TJ:GJ = 100:0:0 obtained the lowest values for Ca (7.37 ± 0.09 mg/100 mL), Mg (5.37 ± 0.07 mg/100 mL), vitamin C (73.60 ± 0.71 mg/100 mL) and β-carotene (67.92 ± 0.76 mg/100 mL), compared to other samples, which showed varied ranges (Ca = from 8.78 to 18.09 mg/100 mL; Mg = from 6.59 to 8.54 mg/100 mL; vitamin C = from 86.74 to 122.97 mg/100 mL; and β-carotene = from 83.19 to 1454.10 mg/100 mL). Increases in Ca, Mg, vitamin C, and β- carotene would most likely be attributed to the addition of turmeric. The vitamin C in fresh turmeric rhizome/root could show very promising levels [10,37,38]. Additionally, the vitamin C in ginger could also show very promising levels [39]. Moreover, the vitamin C in fresh pineapple juice (control) of this current work appeared higher compared with those reported elsewhere, like ~14.1 mg/100 g reported by Ikewuchi and Ikewuchi [32]; 22.5–33.5 mg/100 g reported by Achinewhu and Hart [40]; ~52 mg/100 g reported by Rodríguez et al. [41]; and ~54 mg/100 g reported by Chakraborty, Rao, and Misra [42]. Besides, both vitamin C and β-carotene might not be responsible for the antioxidant capacity of pineapples [40]. Vitamin C would belong to hydrophilic, whereas carotenoids would belong to lipophilic antioxidants [40]. Moreover, the processing of pineapples into juice could likely be affecting, not only quantities of vitamin C [43], but also those of Ca, Mg, and β-carotene contents obtained at this current study.

#### Proximate and Physicochemical Variations

The proximate variations of ginger, pineapple, and turmeric juice mix as influenced by blends can be seen in Table 3. Clearly, the degree of significant differences (*p* < 0.05) were more in the protein, fat, and ash compared to moisture contents across samples. The control PJ:TJ:GJ = 100:0:0 obtained the lowest values for moisture (95.89 ± 0.00%), crude protein (0.008 ± 0.001%), fat (0.051 ± 0.001%), and ash (0.125 ± 0.004%) contents, compared to the blend samples, which showed varied ranges (moisture = from 96.86 to 98.18%; protein = from 0.013 to 0.261%; fat = from 0.061 to 0.168%; ash = from 0.287 to 0.585%). Akusu, Kiin-Kabari and Ebere [4] reported fresh pineapple juice to have about 88% moisture, 1% crude protein, and 2% ash contents, different from values of this current study. Specifically, the increasing amounts of turmeric appears not to dramatically influence the moisture of the blend juice mix, compared to its noticeable influences on the protein, fat, and ash contents. Increases in crude fat and protein might be because of essential oils in ginger and turmeric [10,11]. To the consumer, increases in ash contents portrays the juice mix as a strong mineral source [44]. Ginger and tumeric generally have competitive proximate components, with ranging amounts of 7–13% moisture, 6–12% protein, 60–72% carbohydrate, and 3–7% ash [10,13,14,15]. The marginal influence that increasing tumeric amounts had on moisture might strongly impact on the viscosity of the juice mix blend. Potentially, the blends PTG60:30:10 and PTG50:40:10 respectively with moisture contents of ~98%, would proffer higher sensorial implications compared to the others, especially on both appearance and taste attributes.

The physicochemical variations of ginger, pineapple, and turmeric juice mix as influenced by blends can be seen in Table 4. Clearly, there were significant differences (*p* < 0.05) found in pH, SS, TA, and viscosity across samples. The control PJ:TJ:GJ = 100:0:0 obtained the least values in pH (3.81 ±0.007) and viscosity (300.11 ±0.12 m.Pa.s), but peak values in SS (11.95 ±0.07 °Brix) and TA (0.9005 ±0.07%). Across the blends, noticeable (*p* < 0.05) increases were obtained in pH (from 3.83 to 4.01) and viscosity (from 301.68 to 850.06 m.Pa.s), whereas decreases were obtained in SS (from 9.32 to 4.90 °Brix) and TA (from 0.8425 to 0.5425%). The physicochemical variations from increases in turmeric and decreases in pineapple amounts appear interesting. Increases in pH and viscosity demonstrates the impact turmeric could have in the blend juice mix [45]. Despite the increases in pH and decreases in TA arising from increasing amounts of turmeric [44], the blend mix juice having a peak pH of ~4 would appear somewhat less susceptible to microbial deterioration particularly to the most familiar neutrophilic microorganisms like *Escherichia coli*, staphylococci, and *Salmonella* spp., which are unable to thrive in acidic pH conditions [46]. The decreases in SS might have happened because both turmeric and ginger constituents hold less sugar content(s) compared to those of pineapple [47]. The blends’ viscosity, increasing with quantities of turmeric at this study, might be attributable to its starch [48].

### 3.2. Quality Aspects Microbiological Variations

The microbiological variations of ginger, pineapple, and turmeric juice mix as influenced by blends is shown in Table 5. Across the samples, the bacterial count ranged between 5.0 × 10^3^ and 1.6 × 10^4^ log cfu/mL, whereas the fungi count ranged between 5.0 × 10^3^ and 2.8 × 10^4^ log cfu/mL. The blend PJ:TJ:GJ = 60:30:10 obtained the highest bacterial (1.6 × 10^4^ log cfu/mL) and fungi (2.8 × 10^4^ log cfu/mL) counts. The increases in turmeric reduced the bacterial and fungi counts. The control sample (PJ:TJ:GJ = 100:0:0) obtained the lowest bacterial count, but not so for fungi count. Moreover, the control bacterial and fungi counts both resembled one another (*p* > 0.05). In general, both bacterial and fungi counts were below the microbiological limits prescribed by the Food and Agriculture Organization (FAO) of the United Nations for formulated foods, which is 5 × 10^5^ log cfu/mL, which is largely applicable to both aerobic plate counts (APC) and moulds [49]. The increased turmeric amounts are strengthened by the ginger present, which might have probably brought about the decreases in bacterial counts herein, which points to the antimicrobial capacity (of turmeric).

The antimicrobial inhibition of ginger, pineapple, and turmeric juice mix as influenced by blends, can be seen in Table 6. For emphasis, the test organism was *Salmonella typhii*, and the control used was amoxycillin antibiotics. The result shows that inhibition zones ranged between 12.50 mm (100:0:0) and NSI (No Significant Inhibition) (50:40:10) compared with the control that remained at approximately 20 mm. For emphasis, the control helps to show how the inhibition zone faired compared with those of the blends. Clearly, the antimicrobial activity is depicted by the lowering of inhibition zone as the turmeric was increased. Although the ginger amounts were constant, there is high chance that its presence contributed in strengthening the decreases in the inhibition zones at this study. Spices generally demonstrate antimicrobial activity against bacteria, yeast, molds, and viruses, given its diverse phytochemical components (e.g., alcohols, aldehydes, ethers, hydrocarbons, ketones, as well as phenols), which help to lengthen and stabilize food storage shelf time [10]. Nonetheless, this result goes a step further to demonstrate the presence of active compounds like gingerol, shogaols, and zingerone in ginger, and curcuminoids in tumeric, which provides it with the antimicrobial properties against bacteria such as *Bacillus coagulans*, *B.cereus*, *B. subtilis*, *Escherichia coli*, *Klebsiella pneumoniae*, *Pseudomonas aeruginosa*, *Staphylococcus aureus*, and *S. epidermidis* [10,50,51,52,53].

#### Sensory Variations

The sensory variations of ginger, pineapple, and turmeric juice mix as influenced by blends, can be seen in Table 7. The appearance scores across samples ranged from 7.87 (PJ:TJ:GJ =100:0:0) to 6.73 (PJ:TJ:GJ = 50:40:10). That of taste ranged from 7.27 (PJ:TJ:GJ = 100:0:0) to 3.80 (PJ:TJ:GJ = 50:40:10). Aroma across samples ranged from 7.33 (PJ:TJ:GJ = 100:0:0) to 5.87 (PJ:TJ:GJ = 50:40:10). Mouth feel across samples ranged from 7.40 (PJ:TJ:GJ = 100:0:0) to 4.20 (PJ:TJ:GJ = 50:40:10). General acceptability across samples ranged from 7.60 (PJ:TJ:GJ = 100:0:0) to 5.00 (PJ:TJ:GJ = 50:40:10). Besides, the sensory evaluation has a crucial role to play in judging the quality of a given food product. Control obtained peak values in aroma, mouth feel, and taste, which might have contributed to its lead overall acceptability. Increasing the turmeric and decreasing the pineapple might not necessarily make the blend juice mix more acceptable, given the decreases obtained in appearance, taste, aroma, and mouthfeel scores. Moreover, the sample blend 80:10:10 might be the more preferred compared to the others. Putting together the functional and quality data obtained thus far, we consider the turmeric, ginger, and pineapple blend juice mix nutritionally rich and consumer safe, yet, it might not be generally preferred specifically at this study.

## 4. Conclusions

The functional and quality characteristics of ginger, pineapple, and turmeric juice mix as influenced by blend variations has been determined. The Ca, Mg, vitamin C, and β-carotene contents increased significantly (*p* < 0.05) as quantities of pineapple and turmeric respectively decreased and increased. The degree of significant differences (*p* < 0.05) across samples appeared more in the protein, fat, and ash compared to moisture contents. Despite reducing the bacterial and fungi counts with the inhibition zone, increasing the turmeric and decreasing the pineapple might not necessarily make the blend juice mix more acceptable.

Given the blend results, the nutritional components of the juice mix blends of the current study require further exploration. For instance, the fruit genotype and climatic/storage conditions, together with different geographical regions, could be an influence on functional and quality outcomes of a given juice mix, and this warrants investigation at a future study. Another future work should target to investigate the antioxidant capacity, bioactive components, and total phenolic content of the same juice mix blends. Given the notion that many in Nigeria who take this juice mix consider it nutritionally enriching, future epidemiological and/or economic studies are warranted, as this could help provide additional information that will help substantiate this (notion). Additionally, a direction of future work could also be focused to determine the physicochemical, rheological, quality, and shelf life attributes of this blend juice mix under varying storage conditions.

## Figures and Tables

**Table 1:** Formulation of Ginger, Pineapple, and Tumeric Juice Mix by Blends.

Samples (PJ:TJ:GJ)	Pineapple (mL)	Turmeric (mL)	Ginger (mL)	Total (mL)
100:0:0	100	0	0	100
80:10:10	80	10	10	100
70:20:10	70	20	10	100
60:30:10	60	30	10	100
50:40:10	50	40	10	100

PJ = Pineapple juice; TJ = Turmeric juice; GJ = Ginger juice.

**Table 2:** Minerals and vitamins variations of Ginger, Pineapple, and Tumeric Juice Mix as influenced by Blends.

Samples	Minerals and Vitamin Composition			
PJ:TJ:GJ	Ca (mg/100 mL)	Mg (mg/100 mL)	Vitamin C (mg/100 mL)	β-Carotene (mg/100 mL)
100:0:0	7.37 ^e^ ± 0.09	5.37 ^e^ ± 0.07	73.60 ^d^ ± 0.71	67.92 ^e^ ± 0.76
80:10:10	8.78 ^d^ ± 0.04	6.59 ^d^ ± 0.01	86.74 ^c^ ± 0.23	83.19 ^d^ ± 3.30
70:20:10	12.73 ^c^ ± 0.04	7.67 ^c^ ± 0.02	101.81 ^b^ ± 0.27	199.14 ^c^ ± 1.15
60:30:10	15.63 ^b^ ± 0.19	7.89 ^b^ ± 0.01	103.22 ^b^ ± 0.82	1318.10 ^b^ ± 1.97
50:40:10	18.09 ^a^ ± 0.01	8.54 ^a^ ± 0.01	122.98 ^a^ ± 1.45	1454.10 ^a^ ± 1.69
LSD	0.257	0.081	2.65	5.089

Values are means ± standard deviation (SD) of duplicate determinations. Means in the same column with the same superscript are not significantly different at (*p* > 0.05), Key: PJ = Pineapple juice; TJ = Turmeric juice; GJ = Ginger juice, LSD = Least significant difference.

**Table 3:** Proximate variations of Ginger, Pineapple, and Tumeric Juice Mix as influenced by Blends.

Samples	Tested Parameters (%)			
P:T:G	Moisture	Protein	Fat	Ash
PTG100:0:0	95.89 ^b^ ± 0.00	0.008 ^e^ ± 0.001	0.051 ^e^ ± 0.001	0.125 ^d^ ± 0.004
PTG80:10:10	96.86 ^ab^ ± 1.37	0.013 ^d^ ± 0.001	0.061 ^d^ ± 0.001	0.287 ^c^ ± 0.006
PTG70:20:10	97.91 ^a^ ± 0.01	0.018 ^c^ ± 0.001	0.072 ^c^ ± 0.001	0.322 ^c^ ± 0.001
PTG60:30:10	98.12 ^a^ ± 0.21	0.087 ^b^ ± 0.001	0.101 ^b^ ± 0.001	0.456 ^b^ ± 0.063
PTG50:40:10	98.18 ^a^ ± 0.07	0.261 ^a^ ± 0.001	0.168 ^a^ ± 0.002	0.585 ^a^ ± 0.049
LSD	1.597	0.003	0.007	0.081

Values are means ± standard deviation (SD) of duplicate determinations. Means in the same column with the same superscript are not significantly different at (*p* > 0.05); Key: PJ = Pineapple juice; TJ = Turmeric juice; GJ = Ginger juice; and LSD = Least significant difference.

**Table 4:** Physicochemical variations of Ginger, Pineapple, and Tumeric Juice Mix as influenced by Blends.

Samples	Tested Parameters			
PJ:TJ:GJ	pH	SS (°Brix)	TA (%)	Viscosity (mPa.s)
100:0:0	3.81 ^d^ ± 0.007	11.95 ^a^ ± 0.07	0.9005 ^a^ ± 0.07	300.11 ^d^ ± 0.12
80:10:10	3.83 ^c^ ± 0014	9.32 ^b^ ± 0.028	0.8425 ^b^ ± 0.35	301.68 ^d^ ± 0.78
70:20:10	3.85 ^c^ ± 0.001	8.10 ^c^ ± 0.001	0.6727 ^c^ ± 0.09	351.06 ^c^ ± 1.35
60:30:10	3.89 ^b^ ± 0.014	7.42 ^d^ ± 0.016	0.5775 ^d^ ± 0.21	501.61 ^b^ ± 2.27
50:40:10	4.01 ^a^ ± 0.076	4.90 ^e^ ± 0.004	0.5425 ^e^ ± 0.35	850.06 ^a^ ± 0.06
LSD	0.024	0.081	0.0640	3.178

Values are means ± standard deviation (SD) of duplicate determinations. Means in the same column with the same superscript are not significantly different at (*p* > 0.05), Key: PJ = Pineapple juice; TJ = Turmeric juice; GJ = Ginger juice; LSD = Least significant difference; SS = Soluble Solids; and TA = Titratable Acid.

**Table 5:** Microbiological variations of Ginger, Pineapple, and Tumeric Juice Mix as influenced by Blends.

Samples (PJ:TJ:GJ)	Bacterial Count (log cfu/mL)	Fungi Count (log cfu/mL)
100:0:0	5.0 × 10^3^	5.0 × 10^3^
80:10:10	3.0 × 10^4^	1.0 × 10^4^
70:20:10	1.2 × 10^4^	8.0 × 10^3^
60:30:10	1.6 × 10^4^	2.8 × 10^4^
50:40:10	8.0 × 10^3^	4.2 × 10^3^

Values are means of duplicate determinations. Key: PJ = Pineapple juice; TJ = Turmeric juice; GJ = Ginger juice.

**Table 6:** Antimicrobial inhibition of Ginger, Pineapple, and Tumeric Juice Mix as influenced by Blends.

Samples (PJ:TJ:GJ)	Inhibition Zones of Juice Samples (mm)	Control (mm)
100:0:0	12.50	19.90
80:10:10	12.00	19.90
70:20:10	8.00	19.85
60:30:10	NSI	19.90
50:40:10	NSI	20.00

NSI: No Significant Inhibition. Means of two duplicate determinations. Key: PJ = Pineapple juice; TJ = Turmeric juice; GJ = Ginger juice; The control helps to show how the inhibition zone faired compared with those of the blends.

**Table 7:** Sensory variations of Ginger, Pineapple, and Tumeric Juice Mix as influenced by Blends.

Samples PJ:TJ:GJ	Appearance	Taste	Aroma	Mouth Feel	General Acceptability
100:0:0	7.87 ^ab^	7.27 ^a^	7.33 ^a^	7.40 ^a^	7.60 ^a^
80:10:10	8.27 ^a^	5.87 ^b^	6.67 ^a^	6.20 ^b^	6.33 ^b^
70:20:10	7.73 ^ab^	4.80 ^bc^	6.47 ^a^	5.93 ^b^	5.87 ^bc^
60:30:10	7.93 ^ab^	4.47 ^c^	5.93 ^a^	5.73 ^b^	5.33 ^bc^
50:40:10	6.73 ^b^	3.80 ^c^	5.87 ^a^	4.20 ^c^	5.00 ^c^
LSD	1.27	1.18	1.32	1.15	1.10

Values are means of two duplicate determinations. Means in the same column with the same superscript are not significantly different (*p* > 0.05), Key: PJ = Pineapple juice; TJ = Turmeric juice; GJ = Ginger juice LSD = Least significant difference.

## Data Availability

Data sharing not applicable.
